# Comparison of Clinical Features between Primary Aldosteronism and Essential Hypertension in Chinese Patients: A Case-Control Study

**DOI:** 10.1155/2021/6685469

**Published:** 2021-06-07

**Authors:** Xiaoyu Huang, Shuang Yu, Huangmeng Xiao, Ling Pei, Yan Chen, Wenzhan Chen, Yanbing Li, Haipeng Xiao, Xiaopei Cao

**Affiliations:** ^1^Endocrinology Department, The First Affiliated Hospital, Sun Yat-sen University, Guangzhou, China; ^2^Emergency & Disaster Medicine Center, The Seventh Affiliated Hospital, Sun Yat-sen University, Shenzhen, China; ^3^Pediatric Department, The First Affiliated Hospital, Sun Yat-sen University, Guangzhou, China; ^4^Endocrinology Department, The Seventh Affiliated Hospital, Sun Yat-sen University, Shenzhen, China

## Abstract

Primary aldosteronism (PA) is one of the most common forms of secondary hypertension. Recent studies suggest that, compared with essential hypertension (EH), PA presents more severe disorders of glycolipid metabolism and organ damages. This case-control retrospective study aimed to ascertain clinical features and metabolic parameters between Chinese patients of PA and EH. 174 PA patients and 174 matched EH patients were recruited. Their clinical features, biochemistry parameters, the ventricular septal thickness, and left ventricular mass index (LVMI) were compared. HOMA-*β*% and HOMA-IR were calculated to evaluate glucose metabolism. The results showed that there was no significant difference regarding BMI, waist-to-hip ratio, and blood pressure between the two groups. The blood potassium level was significantly lower in PA patients than those in EH patients. The abnormal glucose tolerance and the incidence of diabetes in the PA group were not significantly different from those in EH group, but the insulin secretion levels at 0 min and 30 min were significantly weaker than those in the EH group, and the HOMA-*β*% was also lower in the PA group than those in the EH group. Left ventricular structural abnormalities in PA patients were more severe than those in EH patients. Subtype analysis indicated that patient with aldosterone-producing adenoma (APA) has more serious hypokalemia and lower levels of HOMA-*β*% and HOMA-IR comparing to those in the idiopathic adrenal hyperplasia (IHA) patient. These findings demonstrated that PA patients showed more impaired insulin secretion function and more severe left ventricular structural damage compared with EH patients.

## 1. Introduction

According to epidemiological surveys, the current prevalence of hypertension in Chinese adults is approximately 27.9% [1]. Hypertension includes primary and secondary hypertension; and secondary hypertension can be caused by conditions that affect the kidneys, arteries, heart, or endocrine system. Primary aldosteronism (PA) is the most common type of secondary hypertension. With the deepening of disease awareness and the improvement of screening efficiency in recent years, PA is found to be a very common type of hypertension, accounting for 5-10% of all hypertensive patients, and even 17%-23% in refractory hypertension [2–4].

PA can be further categorized into aldosterone-producing adenoma (APA), idiopathic adrenal hyperplasia (IHA), primary adrenal hyperplasia (PAH), glucocorticoid-remediable aldosteronism (GRA), pure aldosterone-producing adrenocortical carcinomas, and ectopic aldosterone-secreting tumors [5]. Among these, APA and IHA are the most common clinic types. The main clinical feature of PA is excessive potassium excretion and sodium and water retention that could lead to extracellular fluid expansion and increased blood volume because of the increased secretion of aldosterone. The incidence of hypokalemia in patients with PA is 9% to 37% [6]. In recent years, studies have shown that PA is more common in refractory hypertension and grade 3 hypertension [2]. However, little research has discussed the clinical characteristics of patients with APA versus IHA in Chinese patients.

The relationship between PA and metabolic abnormalities has been reported in 1965 by Conn [7]. Later, a large number of studies in animal and cell models showed that aldosterone affects the body's metabolic processes in terms of insulin secretion, hepatic glycogen uptake and release, and insulin sensitivity. In recent years, several studies indicated that, compared with patients with EH, patients with PA were commonly related to disorders of glucose and lipid metabolism, and they might have more severe damage to targeted organs including heart, brain, and kidney [8–11]. However, there are some studies showing that the impaired glucose tolerance rate and lipid metabolism in patients with PA were more frequent than in healthy individuals, and their prevalence was comparable with the EH patients, giving that the EH patients have a high prevalence of diabetes [11–13]. Therefore, whether there is any difference regarding the metabolic abnormalities between PA and EH in Chinese patients remains to be determined. Moreover, given that the APA and IHA have different etiologies, it is needed to clarify whether the metabolic abnormalities between these two groups are similar.

This case-control retrospective study was carried out to compare the clinical characteristics between PA and EH in Chinese patients. Particularly, the metabolic abnormalities including obesity, dyslipidemia, hyperglycemia, and left ventricular structural damage were discussed.

## 2. Methods

### 2.1. Ethics Statement

The studies involving human participants were reviewed and approved by The Ethical Committee of First Affiliated Hospital of Sun Yat-sen University. All patients provided their written informed consent to participate in this study.

### 2.2. Research Object

A total of 174 patients with PA and 174 patients with EH matched with age, gender, and hypertension course were recruited in the First Affiliated Hospital of Sun Yat-sen University, who were diagnosed from December 2015 to March 2018. Among the 174 PA patients, 111 of them were diagnosed with APA, and 63 were diagnosed with IHA. The included patients were adults between 20 and 80 years old. The exclusion criteria were as follows: (1) patients under 20 or over 80 years old; (2) patients with hypertension caused by other causes, such as renal hypertension, renal artery stenosis, multiple arteritis, pheochromocytoma, or hypercortisolism; (3) patients who had used or were still using diuretics, beta blockers, central alpha 2 blockers, and nonsteroidal anti-inflammatory drugs in the past 2 weeks; (4) patients who had used aldosterone receptor antagonists (such as spironolactone) in the past 4 weeks; (5) patients who had histories of various types of diabetes combined with severe cardiovascular and cerebrovascular diseases, such as severe cardiac insufficiency, ischemic, or hemorrhagic cerebrovascular accidents in the past 1 year; (6) patients who had severe liver or kidney diseases; (7) patients who were pregnant and lactating women; (8) patients who had a history of immune diseases or use of steroid drugs within 3 months; (9) patients suffering from mental illness or using antipsychotics. In addition, twenty-two patients with nonfunctioning adenomas without abnormal glucose, lipid metabolism, and hypertension were recruited for metabolic analysis, serving as a comparison reference when comparing the glucose metabolism between PA patients and EH patients.

### 2.3. PA and EH Diagnosis

The criteria for PA diagnosis and APA or IHA subtype clarification were referred to the 2016 US Endocrine Association guidelines and 2016 China's primary aldosteronism treatment expert consensus [3], with standing ARR as primary screening, with positive intravenous saline load test or positive Captopril challenge test for diagnosis, and CT scan and AVS for subtype clarification.

### 2.4. Blood Biochemistry Test

Blood and urine biochemistry parameters including blood lipids, blood sugar, insulin, and other indicators were collected. The detailed methods of analysis of those parameters are as follows: (1) 24-hour urine retention method: the subject emptied the bladder at 8 : 00 on the first day and began to keep in the urine until the last urination at 8 : 00 the next day. (2) OGTT process: 8 hours after no intake of any heat, morning on an empty stomach, oral 75g anhydrous glucose, dissolved in 250-300 ml water, 5-10 minutes after drinking, fasting, and 30 minutes after starting to drink glucose water, 2 h measured venous plasma glucose, synchronous insulin. (3) venous plasma glucose was determined by hexokinase method; HbA1c was determined by high-pressure liquid phase method; insulin was determined by chemiluminescence immunoassay; total cholesterol (TC) was determined by cholesterol oxidase method (Triglyceride, TG). High-density lipoprotein cholesterol (HDL-c) was measured by the peroxidase scavenging method (SPD method) using the GPO-POD method. Low-density lipoprotein cholesterol (LDL-c) was determined using a surfactant scavenging method (SUR method).

### 2.5. Islet Beta Cell Function and Insulin Resistance Assessment

Insulin initial response index [ΔIRI (30 min)/ΔBS (30 min)]: the ratio of the net increase in blood insulin (ΔIRI) to the net increase in blood glucose (ΔBS) 30 minutes after OGTT. The area under the blood sugar and insulin curve (AAC) is calculated by the approximate trapezoidal area method. The key formulas are as follows:

(1) $$\begin{array}{c} \text{AUC}\left(120\min\right)=\frac{1}{4}*\left(0\min+3*120\min\right)+30\min, \end{array}$$

(2) $$\begin{array}{c} \text{HOMA}-\text{IR}=\text{fasting insulin}\left(\frac{mIU}{L}\right)*\frac{\text{fasting blood glucose}\left(mmol/L\right)}{22.5}, \end{array}$$

(3) $$\begin{array}{c} \text{HOMA}-\beta \%=20\times \text{ fasting insulin}\frac{\left(mIU/L\right)}{\left(\text{fasting blood glucose}\left(mmol/L\right)-3.5\right)}. \end{array}$$

### 2.6. Echocardiography

The comprehensive echocardiographic assessment was conducted by three experienced sonographers, who had no knowledge of the clinical data, using high-quality commercially available ultrasound systems (Philips Ultrasound, Bothell, Washington). All echocardiographic parameters were measured according to the recommendation of the American Society of Echocardiography [14]. LV mass was estimated from the formula of Devereux and Reichek (Penn convention) [15]: LV mass (g) = 1.04 × [(LVDd + IVST + PWT)3-(LVDd)3]-13.6, where LVDd is LV end-diastolic dimension, IVST is interventricular septal thickness, and PWT is posterior wall thickness. The LV mass index (LVMI) was calculated for each subject by dividing LV mass by body surface area.

### 2.7. Statistical Methods

This study used SPSS 25.0 software to perform statistical analysis of all data. The normal distribution measurement data is described by the mean (x) ± standard deviation (SD), and the nonnormal measurement data is described by the median (interquartile range). The *t*-test was used to compare the continuous variables of the normal distribution, the nonparametric rank sum test was used for the nonnormal distribution variables, and the *χ*2 test was used to compare the count data. Correlation analysis used a simple correlation analysis. The *P* value of less than 0.05 was considered statistically significant.

## 3. Results

### 3.1. Comparison of Clinical Features between PA and EH Patients

The baseline data of the two groups were compared. As shown in Table 1, there was no significant difference regarding BMI, waist-to-hip ratio, SBP, and DBP between these two groups, while the waist circumference was higher in EH patients than in patients. The level of electrolytes between patients of PA and EH was compared (Table 1). The blood potassium level of the patients with PA was significantly lower than that of the EH group (3.13 ± 0.77 vs 3.91 ± 0.34, *P* < 0.01), and the blood sodium and urinary potassium levels were higher than those of the EH group (*P* < 0.01). We also observed that the distribution of the blood potassium and sodium levels in these two groups was significantly different. The serum potassium levels of most EH patients were at about 4.00 mmol/L with no cases below 3.00 mmol/L. However, the blood potassium levels of most PA patients were between 1.50 mmol/L and 5.50 mmol/L. The serum sodium distribution of patients in the EH group was 135.00-145.00 mmol/L with no patients exceeding 145.00 mmol/L, while in the PA group, a few cases were seen exceeding 145.00 mmol/L. There was no significant difference in terms of blood and urine calcium levels between these two groups of patients. Despite no significant difference in age, disease duration, gender, blood pressure, and blood lipid profiles, the ventricular septal thickness and LVMI of patients with PA were significantly higher than those of the EH group (*P* < 0.05) (Table 1).

### 3.2. Comparison of Beta Cell Function and Insulin Sensitivity between PA and EH Patients

**Figure 1:**
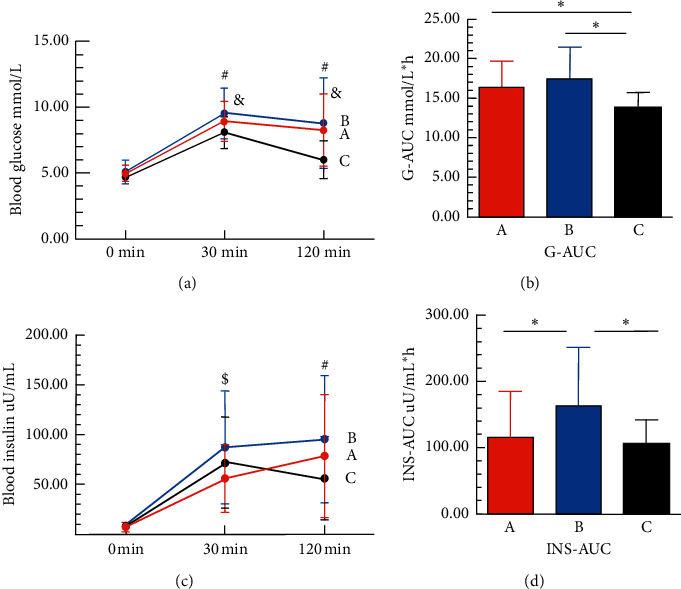
The blood glucose and insulin of OGTT of the three indicated groups. Group A represents patients with primary aldosteronism (PA); Group B represents patients with essential hypertension (EH); Group C represents patients with nonfunctioning adenomas. (a) The curves of the OGTT (0 min, 30 min, 120 min) blood glucose of the indicated group of patients. (b) The AUC of glucose in patients with PA and EH was greater than that in the patients with nonfunctional adenoma. (c) The curves of the simultaneous insulin secretion of the indicated groups of patients. (d) The AUC of insulin in the EH group was significantly higher than that in the other two groups. ^∗^*P* < 0.05; ^#^*P* < 0.05 EH group compared to nonfunctioning adenomas group; ^&^*P* < 0.05 PA group compared to nonfunctioning adenomas group; ^$^*P* < 0.05 EH group compared to PA group.

There were no significant differences regarding the proportion of patients with abnormal glucose metabolism, blood glucose levels of OGTT, and HbA1c between these two groups. However, the OGTT 0 min, 30 min insulin secretion level, insulin initial response index, and insulin curve area in the PA group were lower than those in the EH group (*P* < 0.05). HOMA-*β*% and HOMA-IR were also lower than those in the EH group (*P* < 0.05) (Table 2). We then included another twenty-two patients with nonfunctioning adenomas without abnormal glucose and lipid metabolism and hypertension and drew the curves of the OGTT (0 min, 30 min, 120 min) blood glucose and simultaneous insulin secretion of these three groups of patients (Figure 1). Patients with nonfunctioning adenoma do not have any excessive hormone released into the blood. The data of patients with nonfunctioning adenoma here serves as a reference to determine the effect of aldosterone secretion on glucose metabolism between PA and EH patients. The AUC of glucose in patients with PA and EH was bigger than that in the patients with nonfunctional adenoma. However, no difference was observed in patients with PA versus in patients with EH. In the first 30 minutes, the insulin secretion level of the PA group was the lowest, and the insulin secretion level of the EH group was the highest. The insulin secretion level of the nonfunctional adenoma group showed a downward trend at 30-120 minutes, and the EH group also became gentler than the first 30 minutes. Comparing the AUC of insulin of these three groups, the levels of insulin in the EH group were significantly higher than those in the other two groups (*P* < 0.01) (Figure 1).

### 3.3. Comparison of Clinical Characteristics between APA and IHA Patients

We then further analyzed the 174 PA patients. Among these patients, 111 of them were diagnosed with APA (63.8%) and 63 were diagnosed with IHA (36.2%), as shown in Table 3. The average systolic blood pressure of PA patients was 174.41 ± 23.80 mmHg, and the average diastolic blood pressure was 105.07 ± 14.03 mmHg, and over 60% of patients showed grade 3 hypertension. There was no significant difference in systolic and diastolic blood pressure between APA patients and IHA patients (*P* > 0.05). The average BMI of PA patients was 24.28 ± 4.12 kg/m^2^, and more than half of the patients were overweight or obese. The average body weight, BMI, and waist-hip ratio of IHA patients were not significantly different from those of APA patients (*P* > 0.05), but waist circumference was significantly higher than that of APA patients (*P* < 0.05). Around 63% of PA patients had hypokalemia, with an average blood potassium concentration of 3.13 ± 0.77 mmol/L. The average blood potassium concentration of APA patients was lower than that of IHA patients (2.90 vs 3.54 mmol/L, *P* < 0.01). HbA1c was not significantly different in APA group and IHA group (*P* > 0.05), but HOMA-*β*% and HOMA-IR were higher in IHA patients than in PA patients (*P* < 0.05).

## 4. Discussion

PA is one of the most common causes of secondary hypertension. Hypertension and hypokalemia are the most typical clinical manifestations of PA. This study compared the baseline data and electrolyte levels of PA and EH patients in a case-control manner. Patients in the PA group had lower blood potassium levels than those in the EH group, and urine potassium and blood sodium levels were higher than those in the EH group. This phenomenon could be due to excessive aldosterone secretion acting on aldosterone receptors on the renal tubules and collecting ducts, which alters the permeability of sodium and potassium ion channels, leading to water and sodium retention and increased potassium excretion [16, 17]. In the past, hypokalemia was considered a necessary clinical manifestation of PA. In recent years, with the improvement of diagnostic efficiency, only 9%-37% of patients have been found to have hypokalemia ^4^. In this study, the proportion of PA patients with hypokalemia was 62.60%, higher than some other studies. Given that the patients included in this study were inpatients and their conditions were heavier than those in outpatients, the blood potassium concentration recorded in this study was the same as that of the patients at the first visit. Moreover, patients did not receive any treatments before doing the serum potassium test, so the percentage of patients with hypokalemia was higher than that in other reports.

Previous studies have shown that PA is more likely to be associated with abnormal glucose metabolism such as impaired glucose tolerance and diabetes compared with patients with EH [8, 9, 18], while some other studies hold opposite opinions [12, 13]. In this study, the abnormal glucose tolerance and the incidence of diabetes in the PA group were not significantly different from those in EH group, but the insulin secretion levels at 0 min and 30 min were significantly weaker than those in the EH group, and the HOMA-*β*% was also lower than that in the EH group. The insulin secretion AUC and the initial insulin response index were lower than those of the EH group and the nonfunctioning adenoma group. The above results may suggest that the islet cell secretion function of PA patients could be lower than that of EH patients. However, the lower HOMA-*β*% in PA was not accompanied by other alerted metabolic parameters compared to EH patients. Therefore, although reduced HOMA-*β*% is an index of reduced beta islet cell secretion function, it could not rule out the possibility that PA patients had a better peripheral insulin sensitivity than EH patients. In addition, in previous studies, the IVGTT (iv glucose tolerance test) showed that the first phase (0-10 min) insulin response in the PA group was weaker than that in the EH group [19], which was consistent with our observation.

The effect of excess aldosterone secretion on glucose metabolism is now considered a multifactorial combination. Previous studies have focused on the direct effects of hypokalemia on insulin secretion and subsequently found that even if the patient's serum potassium level is corrected, the patient's abnormal glucose tolerance has not improved. In vitro experiments have found that extracellular potassium can stimulate the release of insulin from cells, so changes in serum potassium can be involved in the regulation of insulin secretion. However, whether it is involved in PA to cause abnormal glucose metabolism remains to be elucidated [20–22]. Previous studies have also suggested that aldosterone can affect the islet secretion function and induce insulin resistance in peripheral tissues through the mineralocorticoid receptor (MR), leading to abnormal glucose metabolism. Moreover, MR activation, in addition to aldosterone, could further promote oxidative stress pathway (increasing NADPH oxidase and ROS levels) and elevate the proinflammatory cytokines (MCP-1, IL-6) in the vascular tissue. The increased oxidative stress and proinflammatory cytokines in turn could lead to impaired insulin metabolic pathway and reduced endothelial-mediated vasorelaxation, eventually contributing to the cardiometabolic change [23].

Recent studies have shown aldosterone mainly through the glucocorticoid receptor (GR) impairing islet *ββ*-cell function rather than the mineralocorticoid receptor, so the use of MR receptor antagonists such as spironolactone may not attenuate the effects of aldosterone on islet *ββ*-cell function in PA [19, 24]. The proportions of glucose metabolism abnormalities between APA patients and IHA patients were comparable. We observed that the values of HOMA-IR and HOMA-*β*% were higher in IHA patients than in APA patients, indicating that islet cell secretion function of APA patients is lower than that of IHA, while insulin resistance is more severe in IHA patients. The mechanisms underlying this difference remain under further investigation. One possible explanation is that IHA is usually diagnosed later than APA, as APA has other features that could lead to early correct diagnosis, such as the young age of presentation and the lower levels of potassium (as confirmed in this study). Also, interestingly, the values of HOMA-IR in IHA patients were very similar to those in EH patients (IHA: 1.92 ± 1.35 versus EH 1.96 ± 0.80), suggesting that although PA patients overall have less severe insulin resistance than EH patients, IHA patients (the subpopulation of PA) seem to have comparable insulin resistance with EH patients. The pathogenetic contributions of aldosterone excess and/or hypokalemia to the glucose metabolism in primary aldosteronism remain to be elucidated. In addition to being prone to abnormal glucose metabolism, studies have shown that patients with PA may have abnormal lipid metabolism, but the results between different studies are not consistent compared with EH patients. Studies suggesting that there may be a correlation between the concentration of aldosterone and certain components of the lipid mass spectrum in the peripheral circulation, including a decrease in HDL-c, an increase in TG and LDL-c [25–29], while other studies showed there was no significant difference in serum lipid mass spectra between the two groups [8, 30]. The mechanism of lipid metabolism disorder in patients with PA may include abnormal insulin metabolism caused by elevated blood pressure, increased insulin levels, and increased gluconeogenesis by TG [31]. In the present study, there was no significant difference in the serum lipid mass spectrometry between the EH group and the PA group. It may be due to the gender, age, and duration of the disease in the study, and the EH patients themselves have a certain degree of insulin resistance.

Previous studies have shown that the incidence of cardiovascular disease, including cardiac insufficiency, myocardial infarction, and atrial fibrillation, in patients with PA is often high, and patients with PA are often associated with changes in cardiac structure, which may be an important cause of cardiovascular accidents [10, 32]. Cardiac structural changes in patients with PA are mainly reflected in left ventricular hypertrophy such as ventricular septal thickening, increased LVMI, and diastolic dysfunction in the heart [26]. In this study, there was no significant difference in blood pressure between two groups. The ventricular septal thickness and LVMI in the PA group were significantly higher than those of the EH group, which was consistent with previous studies [10]. Previous studies have suggested a positive correlation between plasma aldosterone concentration and ventricular septal thickness, and left ventricular posterior wall thickness [33], suggesting that excessive aldosterone secretion has mechanisms that promote cardiac structural changes independent of blood pressure. The mechanism may include aldosterone-induced cardiomyocyte hypertrophy and myocardial fibrosis [32, 34], and after surgical removal of aldosteronism, the left ventricular wall thickness and LVMI of patients may be improved, and the incidence of cardiovascular events is also lower than that before treatment [35].

Also, given that the sodium intake that is usually higher in Chinese population, this dietary factor may be a critical factor that worsened the cardiometabolic changes linked to hypertension in EH patients, leading to higher inflammation levels compared with other different populations.

Several limitations should be noted in this study. First, some important risk factors for cardiac and metabolic abnormalities, such as smoking, dietary preferences, and familiarity with diabetes could be potential confounding variables; and due to missing data, no further analysis was performed. Moreover, several studies have demonstrated the relationship between LVMI and reduced estimated glomerular filtration rate (eGFR) values [36, 37]. We have excluded the patients with severe renal diseases in this study, but whether the patients had mild or moderate renal insufficiency was not further examined. Therefore, the results should be interpreted with caution. Previously, several studies have compared clinical features between PA and EH on the insulin sensitivity and the echocardiography features [38, 39]. Building upon prior publications, this study provides additional information on Chinese patient cohorts with PA and EH. Given that the genetic background and patient dietary preference are different among different populations, the results generated from this study may add additional reference for clinicians when managing different patient populations.

## 5. Conclusion

Our findings confirm a negative effect of aldosterone excess on glucose metabolism and suggest more severe left ventricular structural damage in primary aldosteronism than in essential hypertension. Moreover, the data indicate that the impairment of insulin secretion function is more frequent in PA patients, and the insulin resistance is more severe in EH patients, while among the subtypes of primary aldosteronism, the impairment of insulin secretion function is more frequent in APA patients, and the insulin resistance is more severe in IHA patients.

## Figures and Tables

**Table 1:** The clinic features between PA and EH patients.

	PA group (*n* = 174)	EH group (*n* = 174)	*p*
Age (year)	46.78 ± 11.93	45.71 ± 13.29	0.442
Gender (male/female)	82/94	86/88	0.275
Disease course (year)	4.90 ± 5.76	4.42 ± 4.94	0.419
Body mass index (kg.m-2)	24.28 ± 4.12	25.14 ± 3.80	0.053
Waist (cm)	86.41 ± 11.67	88.40 ± 10.38	0.174
Waist-to-hip ratio	0.90 ± 0.08	0.91 ± 0.07	0.404
SBP (mmHg)	174.41 ± 23.80	175.91 ± 14.03	0.562
DBP (mmHg)	105.07 ± 14.03	106.27 ± 14.20	0.440
Serum K+ (mmol/L)	3.13 ± 0.77	3.91 ± 0.34	<0.001
Urinary K+ (mmol/24 h)	51.95 ± 27.83	33.83 ± 13.78	<0.001
Serum Na+ (mmol/L)	142.02 ± 2.97	140.47 ± 2.09	<0.001
Urinary Na+ (mmol/24 h)	125.07 ± 79.64	107.97 ± 49.88	0.106
Serum Ca2+ (mmol/L)	2.26 ± 0.10	2.28 ± 0.10	0.079
Urinary Ca2 (mmol/24 h)	4.95 ± 2.61	4.81 ± 2.29	0.723
TC (mmol/L)	4.81 ± 1.00	4.87 ± 1.04	0.610
TG (mmol/L)	1.43 ± 0.82	1.52 ± 2.12	0.500
HDL-c (mmol/L) |	2.20 ± 13.33	1.14 ± 0.25	0.319
LDL-c (mmol/L)	3.00 ± 0.71	3.05 ± 0.71	0.523
Septal thickness (mm)	11.21 ± 2.20	10.45 ± 1.76	0.002
LVMI (g/m2)	110.95 ± 35.11	96.21 ± 24.70	<0.001

SBP: systolic blood pressure, DBP: diastolic blood pressure, TC: total cholesterol, TG: Triglycerides, HDL-c: HDL-cholesterol; LDL-c: LDL-cholesterol, LVMI: left ventricular mass index.

**Table 2:** Comparison of glucose metabolic parameters between PA and EH patients.

	PA group (*n* = 174)	EH group (*n* = 174)	*p*
HbA1c (%)	5.66 ± 1.00	5.51 ± 0.56	0.278
0 minBG (mmol/L)	4.97 ± 0.64	5.08 ± 0.88	0.199
30 minBG (mmol/L)	8.92 ± 1.52	9.53 ± 1.94	0.120
2 hPBG (mmol/L)	8.25 ± 2.76	8.79 ± 3.43	0.188
Fasting INS (uU/ml)	7.10 ± 4.66	8.66 ± 3.43	0.030
30 minINS (uU/ml)	55.99 ± 33.90	87.21 ± 56.54	<0.001
2 hINS (uU/ml)	78.34 ± 61.83	95.38 ± 64.04	0.114
G-AUC	16.41 ± 3.27	17.44 ± 4.05	0.055
INS-AUC (uU/ml∗h)	116.19 ± 68.77	162.23 ± 88.32	0.001
ΔIRI/ΔBS (30 min)	13.56 ± 11.68	20.27 ± 15.22	0.004
HOMA-*β*%	98.56 ± 66.70	127.33 ± 78.52	0.023
HOMA-IR	1.62 ± 1.16	1.96 ± 0.80	0.040

BG: blood glucose, INS: insulin, G-AUC: AUC of glucose, INS-AUC: AUC of insulin, ΔIRI/ΔBS: the ratio of the net increase in blood insulin (ΔIRI) to the net increase in blood glucose (ΔBS) 30 minutes after OGTT.

**Table 3:** Comparison of clinic features between APA and IHA patients.

	APA group (*n* = 111)	IHA group (*n* = 63)	*p*
Body mass index (kg·m^−2^)	26.31 ± 5.26	25.35 ± 5.21	0.558
Waist (cm)	84.70 ± 11.31	89.37 ± 12.14	0.016
Waist-to-hip ratio	0.89 ± 0.09	0.92 ± 0.06	0.069
SBP (mmHg)	173.81 ± 24.57	175.48 ± 22.54	0.659
DBP (mmHg)	105.21 ± 12.11	104.82 ± 17.02	0.863
Serum K+ (mmol/L)	2.90 ± 0.73	3.54 ± 0.69	<0.001
Serum Na+ (mmol/L)	142.27 ± 3.19	141.58 ± 2.51	0.144
HOMA-*β*%	84.6 ± 35.36	117.20 ± 91.01	0.028
HOMA-IR	1.40 ± 0.95	1.92 ± 1.35	0.048
HbA1c (%)	5.50 ± 0.66	5.82 ± 1.24	0.285
LVMI (g/m^2^)	114.59 ± 38.67	104.91 ± 27.51	0.107

## Data Availability

The data used to support the findings of this study have not been made available because of protecting the privacy of patients.
